# Treatment-Requiring Postoperative Nausea and Vomiting in Ambulatory Oncology Surgery: Four-Year Trends in Antiemetic Utilization

**DOI:** 10.3390/healthcare14203374

**Published:** 2026-10-10

**Authors:** Jamie Hoffman, Austin Smith, Chris Massengill, Daniel A. Nahrwold, Wendy W. Yang, Raymond Evans

**Affiliations:** Department of Anesthesiology, Moffitt Cancer Center, Tampa, FL 33612, USA; austin.smith@moffitt.org (A.S.); chris.massengill@moffitt.org (C.M.); wendy.yang@stonybrookmedicine.edu (W.W.Y.); raymond.evans@moffitt.org (R.E.)

**Keywords:** postoperative nausea and vomiting, ambulatory cancer surgery, oncology surgery, aprepitant, amisulpride, antiemetics, quality improvement, perioperative outcomes

## Abstract

**Highlights:**

**What are the main findings?**

- Treatment-requiring PACU PONV decreased from 20.3% in 2022 to 9.9% in 2025 across 15,467 ambulatory oncology surgeries.
- Aprepitant was administered in 44.8% of cases, and rescue amisulpride was administered in 4.8% of cases.

**What are the implications of the main findings?**

- Longitudinal quality monitoring demonstrated a marked descriptive decline in treatment-requiring PACU PONV while antiemetic utilization evolved; the aggregate design does not establish causality.
- Patient-level analyses are needed to determine the contributions of baseline risk, case mix, anesthetic practice, prophylactic regimens, and rescue management.

**Abstract:**

Background/Objectives: Postoperative nausea and vomiting (PONV) remains an important complication after ambulatory cancer surgery. This study evaluated four-year trends in treatment-requiring post-anesthesia care unit (PACU) PONV, prophylactic aprepitant utilization, and rescue amisulpride administration. Methods: A retrospective descriptive analysis of monthly perioperative quality-improvement data was conducted from January 2022 through December 2025. The primary outcome was postoperative nausea and/or vomiting requiring antiemetic treatment in the PACU. Because the source data were aggregated, nausea and vomiting could not be analyzed separately, and patient-level risk adjustment was not possible. Results: A total of 15,467 ambulatory cancer surgeries were analyzed. Treatment-requiring PACU PONV occurred in 2041 cases (13.2%) overall and was 20.3% in 2022, 13.3% in 2023, 9.1% in 2024, and 9.9% in 2025. Aprepitant was administered in 6927 cases (44.8%); among aprepitant recipients, 1331 (19.2%) required PACU treatment for PONV. Among 8540 cases without aprepitant, 710 (8.3%) required PACU treatment for PONV. This unadjusted difference should not be interpreted as an estimate of aprepitant effectiveness because treatment allocation was influenced by baseline risk. Rescue amisulpride was administered in 748 cases (4.8%). Conclusions: Treatment-requiring PACU PONV declined over the four-year observation period. Concurrent changes in antiemetic utilization are descriptive and cannot establish that any specific medication or practice change caused the observed decline.

## 1. Introduction

Postoperative nausea and vomiting (PONV) remains one of the most common complications after ambulatory surgery [1,2,3]. Patients undergoing cancer surgery often possess multiple baseline risk factors for PONV, making effective perioperative antiemetic strategies particularly important [4,5].

Aprepitant has demonstrated efficacy in reducing postoperative nausea and vomiting in high-risk surgical populations [1,6]. Despite prophylactic antiemetic administration, some patients continue to experience postoperative nausea and/or vomiting and require rescue treatment in the PACU. Amisulpride has emerged as an effective rescue antiemetic for refractory PONV [7].

The purpose of this study was to describe four-year trends in treatment-requiring PACU PONV, prophylactic aprepitant administration, and rescue amisulpride use among ambulatory cancer surgery patients at a high-volume tertiary cancer center.

## 2. Materials and Methods

A retrospective descriptive analysis was conducted using monthly perioperative quality-improvement data collected from January 2022 through December 2025 at Moffitt McKinley Ambulatory Surgery Center. The project was reviewed by the Moffitt Cancer Center Scientific Review Committee and determined to be a Quality Improvement Project that does not meet the definition of human subjects research under 45 CFR 46; therefore, Institutional Review Board (IRB) approval was not required. The determination was issued on 27 August 2026.

The study population comprised ambulatory cancer surgical cases included in the center’s routine monthly perioperative quality-improvement reporting during the study period. The aggregate dataset did not contain patient-level variables that would permit additional inclusion or exclusion criteria to be applied retrospectively. Monthly variables included surgical case volume, aprepitant administration, postoperative nausea and/or vomiting requiring PACU antiemetic treatment, rescue amisulpride administration, and treatment-requiring PACU PONV among aprepitant recipients. The endpoint was therefore an operational quality metric rather than ascertainment of every episode of nausea or vomiting. Nausea and vomiting could not be analyzed separately.

Aprepitant was preferentially used in patients considered at higher baseline risk for PONV; however, formal risk scores, the specific clinician-level selection criteria, and patient-level risk factors were not available in the aggregate dataset. The dataset also did not capture longitudinal use of other individual prophylactic or rescue antiemetics, including ondansetron and dexamethasone, in a form suitable for analysis. Accordingly, the complete prophylactic regimen, the rescue agents used in cases not receiving amisulpride, whether amisulpride was used as first-line versus later rescue in individual cases, and changes in its position within a rescue algorithm could not be reconstructed. Likewise, procedure type, surgical case mix, anesthetic technique, opioid exposure or opioid-sparing strategies, ERAS pathway participation, and changes in documentation or PACU treatment thresholds were not available for longitudinal analysis.

Descriptive statistics were used to summarize surgical volume, antiemetic utilization, and treatment-requiring PACU PONV. Annual PONV rates were calculated as the proportion of surgical cases requiring PACU antiemetic treatment. For the overall study period, the treatment-requiring PACU PONV rate among cases without aprepitant was derived by subtracting aprepitant-recipient cases and events from the corresponding cohort totals. This comparison is unadjusted and is presented only to describe the aggregate data; no inference regarding aprepitant effectiveness is intended. Temporal patterns were evaluated descriptively; no inferential trend testing was performed, and no claims of statistical significance are made.

Generative artificial intelligence was not used for study design, data collection, or statistical analysis. Its use during manuscript preparation is disclosed in the Acknowledgments Section.

## 3. Results

### 3.1. Study Cohort and Overall Outcomes

A total of 15,467 ambulatory cancer surgeries were included during the study period. Treatment-requiring PACU PONV occurred in 2041 cases (13.2%).

### 3.2. Four-Year Trend in Treatment-Requiring PACU PONV

Annual treatment-requiring PACU PONV decreased from 20.3% in 2022 to 13.3% in 2023 and 9.1% in 2024, followed by a modest increase to 9.9% in 2025. The endpoint therefore remained below 10% in the final two study years, although the pattern was not strictly monotonic. Annual case volume and antiemetic utilization are summarized in Table 1.

### 3.3. Aprepitant Utilization and Treatment-Requiring PACU PONV

Preoperative aprepitant was administered in 6927 cases (44.8%) overall. Among aprepitant recipients, 1331 (19.2%) required PACU treatment for PONV. Among the 8540 cases that did not receive aprepitant, 710 (8.3%) required PACU treatment for PONV. Because aprepitant was preferentially administered to patients considered at higher baseline risk and patient-level risk adjustment was not possible, the higher observed rate among aprepitant recipients should not be interpreted as an aprepitant failure rate or as evidence that aprepitant increased PONV risk.

### 3.4. Rescue Amisulpride Utilization

**Figure 1:**
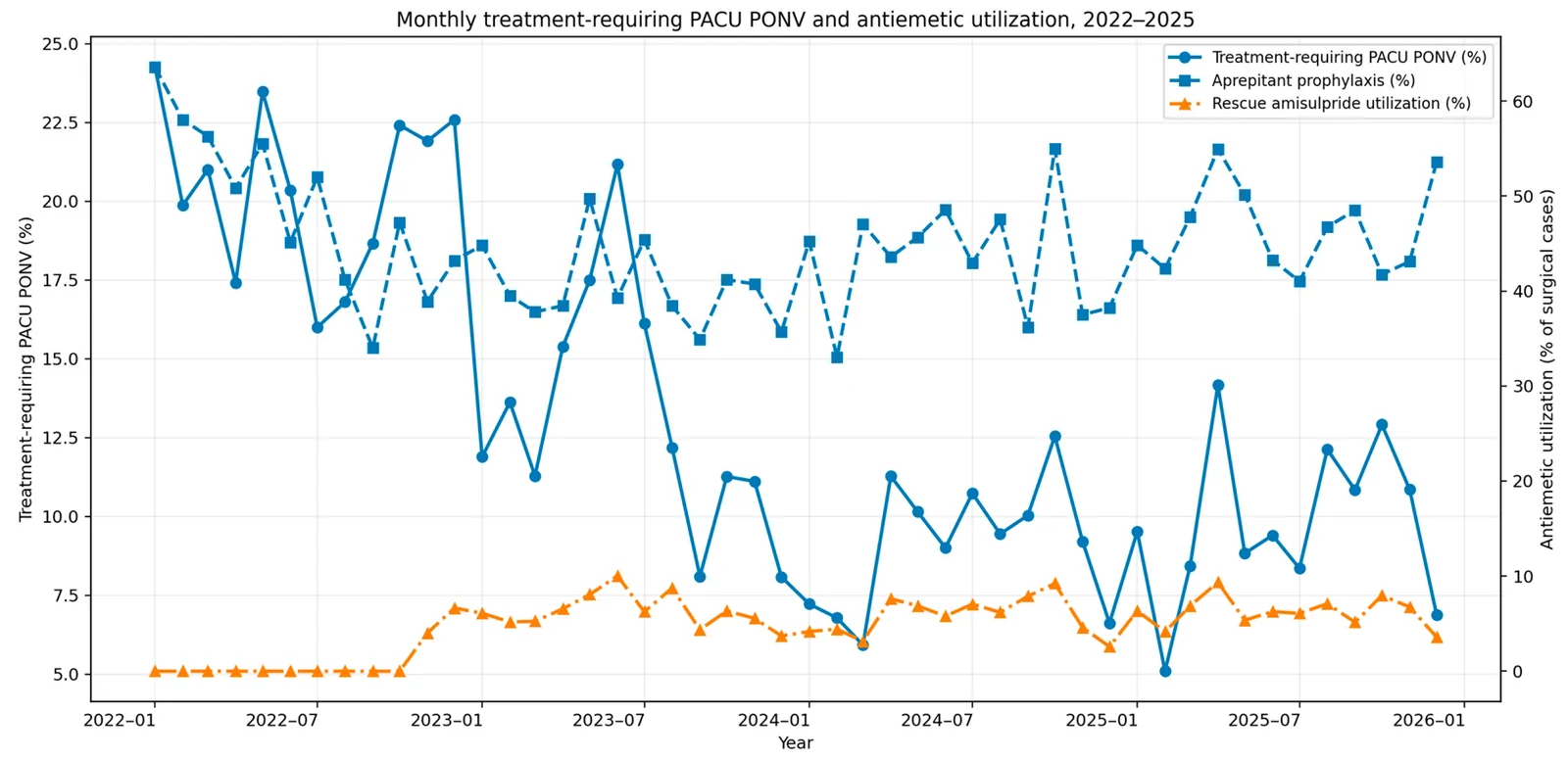
Monthly treatment-requiring PACU PONV and antiemetic utilization from 2022 through 2025. Treatment-requiring PACU PONV (%) is the number of monthly surgical cases with postoperative nausea and/or vomiting requiring PACU antiemetic treatment divided by monthly surgical case volume (left axis). Aprepitant prophylaxis (%) and rescue amisulpride utilization (%) are monthly administrations divided by monthly surgical case volume (right axis). The series use different axes and are descriptive; temporal concurrence should not be interpreted as evidence of a causal medication effect.

Rescue amisulpride was administered in 748 surgical cases (4.8%) during the study period. This variable represents utilization of a rescue treatment and does not measure amisulpride efficacy. The aggregate dataset did not identify the other rescue antiemetics used in the remaining treatment-requiring PONV cases or reliably establish the sequence of rescue agents. Figure 1 displays monthly treatment-requiring PACU PONV and monthly aprepitant and amisulpride utilization. From 2024 to 2025, treatment-requiring PACU PONV increased from 9.1% to 9.9%; over the same interval, aprepitant utilization increased from 43.3% to 46.5%, rescue amisulpride utilization increased from 5.8% to 6.3%, and treatment-requiring PACU PONV among aprepitant recipients increased from 13.8% to 14.7%. These descriptive changes are small and should not be interpreted causally.

## 4. Discussion

This four-year descriptive quality-improvement analysis demonstrated a substantial decline in treatment-requiring PACU PONV among ambulatory oncology surgery patients at a single tertiary cancer center. The annual rate decreased from 20.3% in 2022 to 9.9% in 2025, with the lowest annual rate observed in 2024 (9.1%). The modest increase in 2025 underscores that the pattern was not monotonic. Because no formal trend test or patient-level adjustment was performed, these findings should be interpreted as a descriptive longitudinal pattern rather than evidence of a treatment effect.

Persistent postoperative nausea and vomiting can delay recovery and discharge and negatively affect the surgical experience [1,2,3]. In this study, however, the measured endpoint was specifically postoperative nausea and/or vomiting requiring antiemetic treatment in the PACU. It therefore does not represent all PONV symptoms and may be influenced by symptom burden, treatment thresholds, documentation practices, and PACU workflow.

Aprepitant prophylaxis was used in 44.8% of cases overall. Treatment-requiring PACU PONV occurred in 19.2% of aprepitant recipients and 8.3% of non-recipients in the aggregate data. This unadjusted comparison should not be interpreted as an aprepitant failure rate or comparative effectiveness estimate. Aprepitant was preferentially used in patients considered at greater baseline risk, creating substantial confounding by indication, while formal risk scores and patient-level covariates were unavailable for adjustment [1,6].

Rescue amisulpride was administered in 4.8% of surgical cases. This finding only describes rescue-treatment utilization and does not demonstrate efficacy in this cohort. Evidence supporting intravenous amisulpride as rescue therapy after failed prophylaxis comes from randomized trials outside the present dataset [7]. The aggregate quality-improvement data did not identify all other rescue antiemetics or their sequence, so the role of amisulpride within the institutional rescue algorithm cannot be fully characterized.

The observed decline in treatment-requiring PACU PONV occurred during a period of evolving perioperative practice, but the available dataset cannot identify which concurrent changes contributed. Longitudinal use of ondansetron, dexamethasone, and other prophylactic agents was not available, nor were changes in anesthetic technique, opioid-sparing strategies, ERAS pathways, surgical case mix, patient risk profile, documentation, or PACU treatment thresholds. These unmeasured factors provide alternative explanations for the temporal pattern and preclude attribution of the decline to aprepitant, amisulpride, or any other specific protocol component.

This study has several limitations. It is a single-center descriptive analysis based on aggregated quality-improvement data rather than patient-level clinical variables. Adjustment for sex, smoking status, prior PONV or motion sickness, procedure type, anesthetic technique, opioid exposure, and validated risk scores was not possible. The endpoint combined nausea and/or vomiting and captured only events requiring PACU antiemetic treatment; untreated symptoms and postdischarge events were not measured. The dataset did not permit complete characterization of prophylactic or rescue antiemetic regimens, changes in case mix or ERAS practice, or changes in documentation and treatment thresholds. No inferential trend analysis was performed. Accordingly, causality between evolving antiemetic practices and the observed reduction in treatment-requiring PACU PONV cannot be established.

Future studies using patient-level data, explicit PONV risk stratification, separate nausea and vomiting outcomes, standardized documentation of the complete prophylactic and rescue regimen, procedure and anesthetic characteristics, and postdischarge follow-up would permit adjusted analyses and more definitive assessment of perioperative antiemetic practice.

## 5. Conclusions

In this four-year descriptive analysis of ambulatory cancer surgery, treatment-requiring PACU PONV decreased from 20.3% in 2022 to 9.9% in 2025, with a nadir of 9.1% in 2024. Aprepitant was widely used for prophylaxis and amisulpride was used as a PACU rescue antiemetic. Because treatment allocation, patient risk, case mix, anesthetic practice, other antiemetics, and documentation were not available for adjustment, the observed temporal associations do not establish that any specific antiemetic strategy caused the decline in treatment-requiring PACU PONV.

## Figures and Tables

**Table 1:** Annual treatment-requiring PACU PONV and available antiemetic utilization data.

Year	Surgical Cases, *n*	Treatment-Requiring PACU PONV	Aprepitant Use	PONV Among Aprepitant Recipients	Rescue Amisulpride Use
2022	3887	790 (20.3%)	1896 (48.8%)	513/1896 (27.1%)	33 (0.8%)
2023	3942	525 (13.3%)	1597 (40.5%)	327/1597 (20.5%)	252 (6.4%)
2024	3622	330 (9.1%)	1567 (43.3%)	216/1567 (13.8%)	211 (5.8%)
2025	4016	396 (9.9%)	1867 (46.5%)	275/1867 (14.7%)	252 (6.3%)
Overall	15,467	2041 (13.2%)	6927 (44.8%)	1331/6927 (19.2%)	748 (4.8%)

Values are *n* (%) unless otherwise indicated. Treatment-requiring PACU PONV is calculated using annual surgical case volume as the denominator; aprepitant and amisulpride utilization are calculated as administrations divided by annual surgical case volume; PONV among aprepitant recipients uses annual aprepitant administrations as the denominator.

## Data Availability

The data analyzed in this study were aggregated institutional quality improvement data collected as part of routine clinical operations. Patient-level data were not used for this analysis. The aggregate data are not publicly available. Requests for additional information may be directed to the corresponding author, subject to institutional policies and applicable privacy requirements.

## Abbreviations

The following abbreviations are used in this manuscript:

PONV	Postoperative nausea and vomiting
PACU	Postanesthesia care unit
